# Experiential Course Learning, Wellness, and Higher Education: Qualitative Descriptive Study

**DOI:** 10.2196/88642

**Published:** 2026-05-06

**Authors:** Adrianna Lorraine Watson, Neil E Peterson, Brandon Thatcher, Michael Thomas, Stacie Hunsaker, Cole Hooley, David Erekson, Adam Simpson, Gregory Snow, Rachel Detrick

**Affiliations:** 1 College of Nursing, Brigham Young University, 500 KMBLL, Provo, UT, 84602, United States, 1-801-422-7465; 2 School of Social Work, Brigham Young University, Provo, UT, United States; 3 Student Life, Brigham Young University, Provo, UT, United States; 4 Department of Statistics, Brigham Young University, Provo, UT, United States

**Keywords:** mental health, nursing students, students, health education, health promotion, psychological stress, qualitative research, curriculum, psychological resilience

## Abstract

**Background:**

Undergraduate students, including those preparing for health professions, report high rates of psychological distress and underuse of traditional counseling services. Credit-bearing wellness courses that combine psychoeducation with experiential learning may offer a scalable, curriculum-based approach to supporting student well-being.

**Objective:**

This qualitative study explored how undergraduate students described personal growth, coping, and lifestyle changes following participation in experiential wellness courses.

**Methods:**

An anonymous postcourse online survey captured open-ended responses from students enrolled across 6 wellness course sections. The courses emphasized stress physiology, evidence-based coping strategies, and weekly experiential assignments. Narrative responses from 110 participants were analyzed inductively using the reflexive thematic analysis developed by Braun and Clarke within a constructivist-interpretivist paradigm.

**Results:**

A total of six themes were identified: (1) healthy habits and practical lifestyle change; (2) stress management skills and mental health techniques; (3) self-reflection, awareness, and personal growth; (4) relevance and immediate applicability; (5) peer connection and discussion-based learning; and (6) course structure and opportunities for improvement. Students described adopting new coping strategies, developing greater self-awareness, and perceiving course content as relevant and applicable to their daily lives.

**Conclusions:**

Students described experiential wellness courses as supportive of coping, self-awareness, and behavior change. These findings provide insight into how students engage with and interpret course-based wellness education. Curriculum-integrated approaches may represent a complementary strategy to support student well-being. Future research should examine these approaches across diverse populations and over time.

## Introduction

### Background

Mental health represents a global public health crisis. In 2019, approximately 970 million people were living with a mental disorder [1]. These conditions are shaped by structural determinants, such as food insecurity, violence, and childhood adversity, requiring responses that are context-sensitive and multifaceted [2]. Scalable, accessible approaches that extend beyond specialist care are therefore essential, particularly for young adults who will soon enter and sustain global health, education, and economic systems [3-6].

In the United States, an estimated 59.3 million adults experience mental illness annually, including 36.2% of individuals aged 18 to 25 years [7]. The burden is particularly pronounced among college students. Data from the Spring 2024 American College Health Association National College Health Assessment indicate that 19.5% of students screened positive for serious psychological distress, 25.9% for suicidality, and 48.5% reported loneliness [8]. Despite this, only 25% received counseling in the prior year.

Longitudinal survey data further demonstrate a rising prevalence of depression (21%-38%) and anxiety (22%-34%) between 2014 and 2024, with 13% reporting serious suicidal ideation [9,10]. These trends reflect distress rates comparable to or exceeding those of the general population, alongside persistent underuse of services. Importantly, mental health needs and help-seeking behaviors vary across academic disciplines, underscoring the need for context-sensitive rather than uniform interventions [11].

A range of interventions, including digital platforms, cognitive-behavioral approaches, and mindfulness-based programs, have demonstrated moderate effectiveness in reducing depression and anxiety among university students [12]. However, systematic reviews highlight ongoing challenges, including variability in outcomes, methodological limitations, and issues with engagement and implementation [13]. Earlier work similarly identified gaps in prevention and early intervention strategies within higher education [14]. More recently, attention has shifted toward curriculum-integrated approaches, such as credit-bearing wellness courses that embed mental health promotion within academic environments. Emerging evidence suggests these interventions may improve stress, coping, and help-seeking behaviors [15], yet existing studies are often limited to single courses or specific populations and provide limited insight into student experience or mechanisms of impact [16-19]. This gap is particularly relevant given rising student distress and continued underuse of traditional services. To address this, this study explored undergraduate students’ experiences of experiential wellness courses using a descriptive qualitative design with an inductive approach.

### Aim

The purpose of this study was to explore students’ experiences and the perceived effectiveness of experiential wellness courses. The research question guiding this inquiry was: How do undergraduate students describe their experiences, the perceived effectiveness, and the personal impact of experiential wellness courses?

## Methods

### Qualitative Approach and Research Paradigm

#### Reflexive Thematic Analysis

Narrative data were analyzed using the 6-phase reflexive thematic analysis (familiarization, coding, theme development, review, definition, and reporting) developed by Braun and Clarke [20]. This inductive approach supported repeated movement between the full dataset and individual meaning units, allowing patterns to be identified from participants’ language while preserving interpretive depth. First-cycle coding was conducted manually, with analysts working line-by-line through the data using a flexible combination of in vivo and descriptive codes. In some excerpts, analysts also applied more than one code where multiple meanings were present. Codes were then discussed and iteratively refined through reflexive dialogue, memo writing, and repeated comparison across responses as themes developed. Table 1 summarizes the primary coding practices used in the early stages of analysis.

**Table 1.** Illustrative first-cycle coding practices^a^.

Coding practice	Analytic function	Example
In vivo (participant-language) coding	Preserved participants’ wording and stayed close to lived experience	“Feeling in control”
Descriptive coding	Summarized the main topic, action, or experience in a segment	“Improved sleep habits” and “time management strategies”
Simultaneous coding	Captured more than one meaning within a single excerpt	Same excerpt coded as coping skill use and identity shift

a These coding practices were used flexibly and interpretively rather than as fixed, mutually exclusive categories*.*

#### Paradigm

The study was situated within a constructivist-interpretivist paradigm, which assumes that meaning is coconstructed and context-dependent. Accordingly, the analysis focused on interpreting the range and depth of student experiences rather than quantifying response frequency. Within this approach, researcher subjectivity was understood as a resource for interpretation rather than a source of bias to be minimized.

#### Rationale

This approach aligns with the study’s aim of understanding how students interpret and apply experiential wellness curricula. Reflexive thematic analysis provides a flexible yet systematic method for examining meaning-making processes related to coping, behavior change, and personal development.

### Researcher Characteristics and Reflexivity

The analytic team brought complementary clinical, multidisciplinary, and lived perspectives to the study. The research team consisted of faculty researchers and student research assistants with backgrounds in nursing, social work, counseling psychology, and statistics. Recognizing that their professional roles could shape data interpretation, team members engaged in continuous reflexive practice. Before analysis, each analyst recorded positionality statements detailing personal wellness beliefs, prior exposure to college-health settings, and expectations about course impact. These statements were revisited during peer-debrief meetings to surface implicit assumptions. Differing interpretations were explored through reflexive discussion, with attention to how analytic perspectives were shaped by researchers’ disciplinary and experiential backgrounds [20,21].

### Context and Sampling Strategy

The study was conducted across 6 undergraduate wellness courses offered over 4 semesters (2021‐2022 and 2022‐2023) at a private university in the Western United States. The courses were semester-long (14‐16 weeks), credit-bearing electives, and varied in topical focus, including mindfulness-based wellness, stress management and resilience, positive psychology, cognitive and behavioral wellness, lifestyle behavior change, and integrative wellness or student development. Course content emphasized stress physiology, cognitive restructuring, mindfulness practices, behavior change strategies, and intentional self-care, with instruction delivered through weekly applied assignments, structured reflection (eg, journaling), and discussion-based learning. Table 2 summarizes the courses, including target learners, educational objectives, enrollment size, instructional format, and core content areas.

**Table 2.** Characteristics of experiential wellness courses

Course type or focus area	Target learners	Educational objectives	Enrollment size (approximately)	Instructional format	Core content areas
Mindfulness-based wellness	Undergraduate students (mixed majors; includes nursing/prenursing)	Develop awareness of stress responses and cultivate mindfulness-based coping strategies for emotional regulation	15‐25	Small-group, discussion-based, and experiential	Mindfulness practices, breathwork, present-moment awareness, and stress reduction
Stress management and resilience	Undergraduate students (health and nonhealth majors)	Build practical stress management skills and enhance resilience for academic and personal challenges	20‐30	Hybrid (lecture, experiential, and discussion)	Stress physiology, coping strategies, relaxation techniques, and resilience-building
Positive psychology and well-being	Undergraduate students (mixed majors)	Promote well-being through strengths-based approaches and meaning-making strategies	20‐30	Discussion-based with reflective activities	Gratitude, strengths identification, purpose, and emotional well-being
Cognitive and behavioral wellness	Undergraduate students (including prehealth students)	Apply cognitive and behavioral strategies to improve thought patterns, emotional regulation, and behavior change	20‐30	Hybrid (lecture and applied practice)	Cognitive restructuring, thought patterns, behavior modification, and self-regulation
Lifestyle and health behavior change	Undergraduate students (including exercise science and prehealth students)	Support development of sustainable health behaviors and habit formation	20‐35	Experiential learning with weekly assignments	Sleep hygiene, time management, physical activity, and habit formation
Integrative wellness or student development	General undergraduate population (elective and nonhealth specific)	Encourage holistic self-awareness, personal development, and interpersonal well-being	15‐25	Discussion-based	Self-reflection, goal setting, peer dialogue, and holistic wellness

Participants were a heterogeneous undergraduate sample that included nursing and prenursing students, other health-related majors (eg, exercise science), and nonhealth profession students. The courses functioned both as general education electives and as preparatory or complementary learning experiences for students pursuing health professions. A purposive sampling strategy was used to capture variation across pedagogical approaches, topical emphases, and disciplinary contexts within the university’s wellness curriculum. All registered students in the 6 selected courses were invited to complete an anonymous end-of-semester Qualtrics survey with open-ended prompts. Inclusion criteria were age 18 years or older, English fluency, enrollment in 1 of the 6 target courses, and completion of at least 1 substantive open-ended response. Surveys with blank qualitative items were excluded. Because data were collected from a fixed set of courses, formal thematic saturation was not used as a stopping criterion. Instead, sampling prioritized conceptual breadth across course types to support transferability and the identification of cross-cutting patterns in student experience.

### Ethical Considerations

The Brigham Young University Institutional Review Board ethically approved this study (IRB2021-192). Participants provided informed consent, and responses were deidentified on the online platform. Incentives for completion of the survey consisted of a US $5 gift card.

### Data Collection Methods

Data were collected via an anonymous postcourse online survey administered through Qualtrics after course completion. All enrolled students were invited via email, and participation was voluntary. Of the 125 consenting students, 110 (88%) provided usable responses. The survey system restricted submissions to one per participant, and no duplicate entries were identified. Survey design and reporting adhered to guidelines for web-based research [22].

### Data Collection Instruments and Technologies

The research team developed open-ended survey prompts informed by literature on college student mental health, wellness education, and experiential learning. The prompts were designed to elicit perceptions of course effectiveness, engagement with experiential components, and perceived impact on well-being, including coping strategies, behavioral change, self-awareness, and personal growth. The instrument was developed collaboratively by a multidisciplinary team and was iteratively reviewed for clarity, relevance, and alignment with study aims. Consistent with the exploratory qualitative design, the instrument was not pilot tested or psychometrically validated.

### Data Processing

Survey responses were exported to a secure, encrypted file. Identifying information was removed, and responses were assigned numeric codes. Data were organized in Microsoft Excel to facilitate coding and analysis.

### Data Analysis

Analysis was conducted using reflexive thematic analysis [20]. A total of 2 analysts engaged in iterative, reflexive coding of the dataset, initially working through the data individually and then meeting regularly to discuss interpretations, explore alternative readings, and refine the evolving analytic framework. Coding was inductive and conducted line-by-line, using a flexible combination of in vivo (participant-language) and descriptive codes, with occasional simultaneous coding where excerpts reflected multiple meanings (Table 1).

Codes were not treated as fixed entities but were developed and refined through ongoing engagement with the data, supported by memo writing and an audit trail documenting analytical decisions. Through iterative comparison across responses, codes were grouped into candidate themes, which were subsequently reviewed and refined to ensure internal coherence and conceptual distinction. Themes were then defined and illustrated using representative participant quotations. Consistent with reflexive thematic analysis, the analytic process emphasized reflexivity, interpretive depth, and the coconstruction of meaning, rather than coding consensus or reliability. Reflexive journaling supported ongoing attention to how researchers’ perspectives shaped interpretation throughout the analytic process.

### Techniques to Enhance Trustworthiness

To enhance rigor, triangulation was operationalized through an iterative comparison of three sources: (1) student narrative responses, (2) relevant contemporary literature on wellness education and mental health interventions, and (3) input from external content experts. During analysis, the research team engaged in ongoing cycles of comparison between emerging codes and themes and existing literature. As preliminary patterns were identified, analysts conducted targeted literature checks to examine whether these patterns aligned with, extended, or diverged from prior findings. These comparisons were documented in analytic memos and discussed during regular team meetings, allowing for the refinement of theme definitions and ensuring that interpretations were both grounded in participant accounts and situated within the broader evidence base.

External expert review was incorporated after the initial theme development. A total of 2 subject-matter experts in counseling psychology and health promotion were provided with a summary of candidate themes, representative excerpts, and the analytic rationale. The experts were asked to assess conceptual clarity, coherence, and alignment with current knowledge in the field. Their feedback was discussed by the analytic team and used to refine theme boundaries, naming, and interpretation where appropriate. All changes informed by expert input were documented in the audit trail. This triangulated approach strengthened the credibility and confirmability of the findings by ensuring that interpretations were grounded in participant narratives while also being critically examined through both scholarly and expert perspectives.

## Results

### Participant Characteristics

Of the 125 students enrolled in wellness courses who consented to participate, 110 (88%) provided usable postcourse responses for analysis. Demographic information was available for the 125 consented participants. These participants were drawn from 6 courses offered across 4 semesters (2021‐2022 and 2022‐2023 academic years), representing a range of disciplines, including exercise science, interdisciplinary general education (honors), nursing, and student development; the latter reflects a general undergraduate or student affairs context rather than a health professions program (Table 3). Of the 125 students, the sample was predominantly female (n=115, 92%) and non-Hispanic White (n=113, 90.4%). Participants were relatively evenly distributed across academic years: first year (n=28, 22.4%), second year (n=32, 25.6%), third year (n=26, 20.8%), and fourth year (n=26, 20.8%), with a smaller proportion in their fifth year or beyond (n=13, 10.4%).

**Table 3.** Participant characteristics of consented students

Characteristic	Value, n (%)^a^
Total consented participants	125 (100)
Provided usable postcourse responses for qualitative analysis	110 (88.0)
Sex	
Female	115 (92.0)
Male	10 (8.0)
Race/ethnicity	
Non-Hispanic White	113 (90.4)
Other race/ethnicity	12 (9.6)
Academic year	
First year	28 (22.4)
Second year	32 (25.6)
Third year	26 (20.8)
Fourth year	26 (20.8)
Fifth year or beyond	13 (10.4)

a Percentages are based on the total consented sample and may not equal 100 due to rounding.

### Emergent Themes

#### Overview

**Figure 1.**
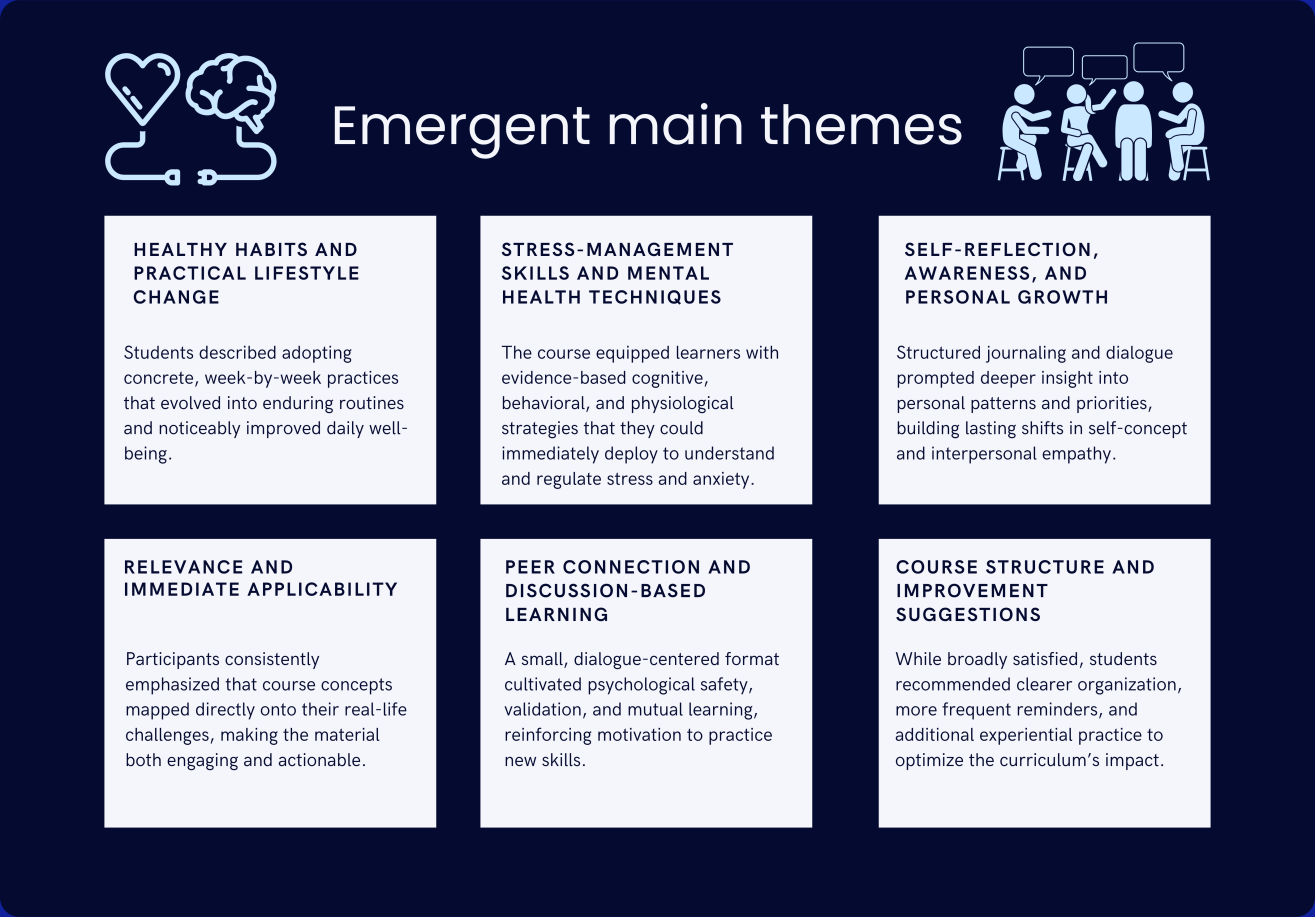
Overview of main themes.

Analysis revealed six emergent main themes: (1) healthy habits and practical lifestyle changes; (2) stress management skills and mental health techniques; (3) self-reflection, awareness, and personal growth; (4) relevance and immediate applicability; (5) peer connection and discussion-based learning; and (6) course structure and opportunities for improvement. See Figure 1 for a brief overview of these themes. The 6 courses described above represent the instructional context from which participants were sampled; however, the themes presented below reflect cross-cutting patterns identified across all participants and course types. Themes did not map to individual courses but instead captured shared experiential outcomes that emerged across varied instructional formats, content areas, and student populations. The analysis, therefore, focuses on common patterns in how students described the impact of experiential wellness learning, rather than on course-specific differences.

#### Theme 1: Healthy Habits and Practical Lifestyle Change

Participants across courses described their experiences in wellness courses as a catalyst for tangible, day-to-day transformation. Rather than learning abstract principles, they reported adopting new behaviors, routinizing them, and noticing measurable gains in well-being. One student captured the overarching impact succinctly: “It helped encourage healthy life habits that I saw having a large improvement in my life.” The perception that change was both visible and personally meaningful was evident in every account.

A central driver of this shift was the rhythm of experiential homework. Weekly assignments were not viewed as traditional coursework but as opportunities to “learn how to be a happier person.” Students repeatedly emphasized that these tasks translated concepts into action; by “consciously set[ting] apart time every week to help destress,” they could evaluate strategies in real contexts and refine them over time.

Learners also spoke of accumulating skills in a deliberate, scaffolded sequence. As 1 participant explained:

*We covered a new topic each week to focus on and apply into your life. It [built] on each other so that by the end of the semester, I had a lot of good new habits.*

This progressive layering fostered both competence and confidence, allowing practices to migrate from structured exercises to authentic habits embedded in daily routines.

Crucially, the motivation to change was experienced as self-endorsed. Students highlighted an “emphasis on growth for yourself and changing your lifestyle because you want to,” describing the activities as “realistic, practical ways to be and feel better.” Far from being externally imposed requirements, the assignments were perceived as invitations to experiment with well-being strategies and to keep what worked.

The tangible nature of the outcomes fortified engagement. As 1 learner reflected, “I liked putting into practice what we learned and seeing for myself the difference it made.” Another credited the course grading structure for sustaining momentum, “I think the experiential assignments were most helpful too because we had motivation to reach our positive living goals since it was for a grade.” Grades functioned less as extrinsic pressure than as a supportive scaffold, prompting sustained practice until new behaviors took root.

By the close of the term, many participants articulated a sense of ownership over their emerging lifestyles. One student concluded, “I loved the skills and habits I developed after taking the class,” signaling that the practices had become woven into the fabric of everyday life. Narratives reveal a pattern of intentional, self-directed habit formation grounded in weekly experiential work, which yielded authentic, lived improvements in wellness.

#### Theme 2: Stress Management Skills and Mental Health Techniques

Students described the course as a practical workshop in evidence-based coping methods. Far from remaining abstract, concepts were embedded in hands-on activities that allowed immediate application. One participant captured this ethos by noting they “liked being able to try different stress-relieving activities,” while another emphasized mastery of “simple skills to help me cope with stress… it has helped me so much.” Across accounts, learners framed stress not as an unavoidable burden but as a challenge that could be actively managed with the right tools.

A recurring narrative centered on biological and cognitive insight. Several students highlighted that understanding “how the brain works” or “how the autonomic nervous system works and why” fundamentally shifted their appraisal of anxiety and pressure. This psychoeducation component laid the groundwork for skill adoption, “Learning some stress physiology and practical skills to manage stress, plus a shift in how I think about stress,” signaled that knowledge and practice were mutually reinforcing.

Participants described a diverse toolkit that spanned cognitive, behavioral, and mindfulness-based techniques. Cognitive restructuring, time-management strategies, and relaxation exercises emerged as particularly salient. One student summarized the breadth of learning:

*The most helpful things were learning about cognitive restructuring, learning about how the stress response works, and time-management skills. And relaxation skills.*

Another underscored the power of targeted practice, “We practiced simple techniques to acknowledge and cope with intrusive thoughts.”

For some, the techniques proved strikingly consequential. The statement that “defusion has literally been a lifesaver and stopped me from cutting multiple times” points to the clinical potency of even brief interventions when they are delivered in an autonomy-supportive, experiential format. Collectively, student narratives suggest a progression: (1) students gained conceptual clarity about stress, (2) rehearsed concrete strategies in low-stakes settings, and (3) finally generalized these skills to real-world contexts where stress and anxiety naturally arise.

Learners framed these practices as foundational life capacities rather than course-bound tasks. They anticipated ongoing relevance because stress is “bound to be present” in adult life, positioning the acquired techniques as durable resources. The course, therefore, functioned as both an introduction to and a rehearsal of coping strategies, empowering students to meet future challenges with a richer, more specialized toolkit.

#### Theme 3: Self-Reflection, Awareness, and Personal Growth

A third strand of participant experience centered on deepened self-understanding. Learners consistently portrayed the course as a mirror that helped them see themselves (and others) with new clarity. One student remarked*:*

*I learned so much about myself and how to better my own mental health. I’ve noticed a difference in how I approach problems and I will absolutely take what I’ve learned with me in the future.*

This forward-looking sentiment illustrates how insights gained in class were immediately translated into everyday decision-making.

Structured reflection activities invited students to identify priorities and patterns. The journal exercises, for instance, enabled 1 participant to “self-reflect on my life and see what my priorities are,” while small-group dialogue allowed another to “open up,” describing the process as an opportunity that “gave me new perspective on things.” Such revelations frequently extended beyond the individual to a relational sphere; several students reported learning “how to identify personal signs of struggling with mental health as well as how I can recognize when others are struggling,” underscoring heightened interpersonal awareness.

The personal nature of the coursework also fostered a sense of direction. One learner explained that the class “helped me figure out what I specifically want to work out in myself. It gave me direction,” pointing to emergent goal-setting rooted in self-diagnosis. For others, customized assignments enhanced engagement, “My favorite part was how personalized it was...the way that the assignments catered to me individually to help me develop my habits was really cool and rewarding.” Emphasis on tailoring appears to have strengthened both motivation and perceived relevance.

These narratives reveal that students began by examining internal states, progressed to recognizing behavioral patterns, and ultimately reoriented future actions in light of these insights. In the words of 1 participant, “The experience really improved how I view myself, others, and the world around me,” signaling a broadened lens that blended self-care with empathic attunement to the well-being of others.

#### Theme 4: Relevance and Immediate Applicability

A concise but recurring narrative highlighted how readily students could transfer course material into everyday life. One participant summarized the sentiment in just 2 words: “relatable” and “applicable.” Rather than perceiving the curriculum as theoretical or distant, learners repeatedly emphasized its direct fit with their current challenges and goals. As 1 student noted, “The concepts we learned about were super applicable and relevant to my life,” underscoring ideas introduced in class that mapped neatly onto ongoing personal circumstances.

Several accounts pointed to a sense of novelty coupled with practicality. A participant celebrated the “fresh perspective it offered on happiness,” adding that this outlook was “applicable...to my life.” The description implies that relevance was not merely a function of content overlap but of insight; students encountered familiar struggles reframed through new, actionable lenses. Across comments, the prevailing tone suggested that applicability heightened motivation; when strategies resonated with real‐world contexts, learners became more willing to test them and, by extension, sustain engagement with the course.

#### Theme 5: Peer Connections and Discussion-Based Learning

A prominent feature that shaped students’ experiences was the interpersonal fabric of the classroom. Participants did not merely learn about well-being; they learned with and through one another, crediting the small, dialogue-oriented format for amplifying insight and comfort. One learner captured this dynamic succinctly: “Getting to know my peers, talking as a group.” Repeatedly, students described the course as “discussion based,” emphasizing that conversation, rather than lecture, was its heartbeat.

Across courses, smaller class sizes appeared to support a more intimate scale, which participants perceived as allowing everyone to speak and be heard. For example, 1 participant called it “an open dialogue with my peers.” Another echoed this sentiment, noting they “loved having a class that was small where the students were able to interact frequently with one another.” Such proximity translated into psychological safety; learners reported that the setup “helped me participate more than I usually would,” *s*uggesting that the environment lowered barriers to sharing personal struggles and successes.

The relational tone of the course appeared to satisfy a need for validation and empathy. One student explained, “Discussions helped me feel understood and heard and helped me relate to other people,” highlighting the reciprocity inherent in storytelling and listening. Another participant emphasized the value of mutual disclosure, stating that “Discussing with others and sharing my experiences when possible was very helpful and validating for me.” This validation intensified the perceived relevance of the course content; hearing peers navigate similar challenges reinforced the applicability of the strategies being taught.

Peer connection was not an ancillary benefit but a central learning mechanism. Dialogue provided a richer context for theoretical concepts, encouraged reflection, and built a supportive community where new habits could take root. In this way, the social architecture of the course both mirrored and strengthened well-being objectives, demonstrating that personal growth is often cultivated most powerfully in the company of others.

#### Theme 6: Course Structure and Opportunities for Improvement

Alongside widespread enthusiasm, students offered constructive responses regarding the course’s scaffolding. The most common critique concerned organizational clarity. One participant remarked, “There was a lack of direction, which led to a lack of organization. I would suggest a more detailed curriculum plan,” signaling a desire for clearer road-mapping of weekly expectations and major assignments. Others focused on operational details that would streamline engagement; several learners asked instructors to “send out reminders during the week for our different ‘challenges’” because those tasks were “easy to forget.”

A second cluster of comments advocated for expanded experiential practice. Although students valued the hands-on components, some felt the dosage could be higher:

*More experiential homework assignments. The practice is one of the most important outcomes off this class. The more practice we get, the more ways we can see implementation and start to create habits.*

This feedback suggests that the experiential spiral highlighted in earlier themes could be strengthened by allocating additional time or credit to real-world application.

Learners also imagined ways to broaden the course’s reach. One participant urged, “I would invite more people to join. They really do not know what they are missing,” while another proposed shifting focus to “how to make your smaller community flourish and not just a bigger institution,” hinting at an interest in service-oriented or peer-mentoring extensions.

Notably, praise and critique co-existed. Even students who recommended tweaks often emphasized overarching satisfaction:

*I honestly don’t think I would change anything about the course. It was planned well and the instructor did a great job helping us learn material and giving us space to discuss amongst ourselves.*

Participants sought fine-tuning (more transparent structure, timely reminders, and additional practice opportunities) rather than an overhaul, while affirming that the existing framework already delivered meaningful growth.

### A Proposed Model of Undergraduate Wellness: “The Reflect-Recognize-Reorient Cycle*”*

**Figure 2.**
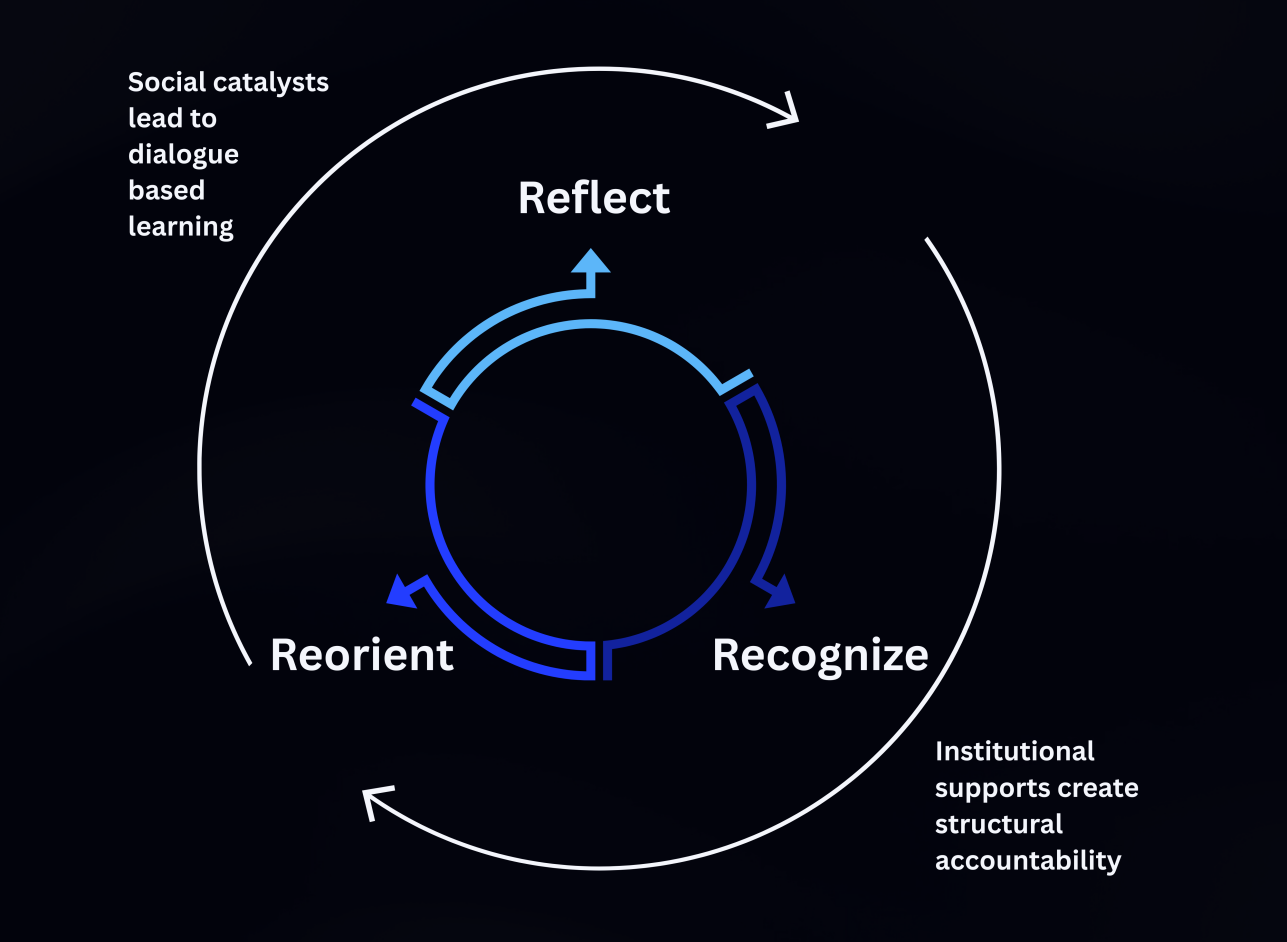
The reflect-recognize-reorient (R^3^) cycle.

Across themes, students described an iterative 3-part process that recurred throughout the semester (Figure 2).

#### Phase 1: Reflect

Students first turned their attention inward, identifying thoughts, emotions, and bodily cues they had previously overlooked. This mirrors the Kolb reflective observation and aligns with the Mezirow emphasis on critical self-reflection, yet our data foreground a distinctly affective, mindfulness-based stance that is not fully elaborated in either classical model [23-26].

#### Phase 2: Recognize

Having surfaced their internal states, students began to identify recurring behavioral patterns and situational triggers (“I spiral every time a deadline looms*”*). Conceptually, this bridges the Kolb abstract conceptualization and Mezirow meaning-making, but the reflect-recognize-reorient (R^3^) cycle positions pattern recognition as a discrete, sense-making pivot rather than a by-product of reflection [23-26].

#### Phase 3: Reorient

Finally, students formulated concrete, forward-looking actions: scheduling micro-breaks, seeking peer support, or practicing paced breathing before exams. This corresponds to the Kolb active experimentation and the Mezirow reintegration; however, our participants described reorientation as value-driven, often linking new actions to a broader life purpose that surfaced in phases 1‐2 [23-26].

## Discussion

### Principal Findings

Findings suggest that undergraduate students’ experiences and perceived effectiveness of experiential wellness courses are characterized by 6 cross-cutting themes that illustrate how these courses may support student well-being. In this study, participants describe meaningful changes in daily behaviors, including the development of healthier habits and the application of practical coping strategies such as mindfulness, cognitive restructuring, and time management. In addition to skill acquisition, participants report increased self-awareness and personal growth, often accompanied by shifts in how they conceptualize stress, success, and self-care. Students consistently emphasize the immediate relevance and applicability of course content, which appears to enhance engagement and the sustained use of strategies. Peer interaction and discussion-based learning emerge as central mechanisms that foster validation, connection, and motivation. Although participants identify opportunities to improve course structure and organization, the findings collectively indicate that experiential, course-based wellness education supports both behavioral change and deeper meaning-making processes related to mental health and well-being.

### Comparison With Literature

Alignment with and extension of existing research on mental health interventions suggest that participants may report improvements in well-being following exposure to structured interventions. For example, meta-analytic evidence demonstrates that cognitive-behavioral and mindfulness-based approaches produce moderate reductions in depression and anxiety among college students, and participants in this study similarly reported acquiring practical skills such as cognitive restructuring, relaxation techniques, and time management strategies [12]. These findings reinforce the effectiveness of skill-based approaches to mental health promotion within undergraduate populations.

The findings also extend current understanding by highlighting the importance of experiential and contextually embedded delivery. Previous research indicates that mental health interventions, particularly digital and self-guided formats, often encounter challenges related to engagement, uptake, and sustained use [13]. In contrast, participants in this study emphasize that embedding wellness strategies within structured, credit-bearing courses enhances both engagement and accountability. The integration of weekly experiential assignments and reflective activities appears to support sustained behavior change, suggesting that curriculum-based approaches may mitigate implementation challenges identified in prior research.

The findings further extend earlier work on prevention and early intervention in higher education. Foundational reviews identify a limited and inconsistent evidence base for preventive mental health interventions among university students, particularly for anxiety and depression [14]. Although recent studies demonstrate increased interest in course-based and curriculum-integrated approaches, much of the existing literature remains focused on discrete interventions or single-course implementations. Recent scoping reviews further highlight that while well-being interventions are increasingly implemented across higher education, many lack theoretical coherence and are not systematically integrated into academic curricula [27]. By examining student experiences across multiple wellness courses, this study identifies cross-cutting patterns of change, including the development of durable coping skills, increased self-awareness, and shifts in meaning-making.

The prominence of self-reflection, peer dialogue, and perceived relevance in these findings adds important nuance to the intervention literature. Prior studies primarily emphasize symptom reduction and measurable psychological outcomes, whereas participants in this study describe changes at both behavioral and identity levels. These findings suggest that wellness courses function not only as skill-building interventions but also as environments that support relational learning and meaning reconstruction. In doing so, the study bridges intervention-based research and transformative learning theory, offering a more integrated account of how mental health promotion occurs within educational settings.

Finally, the findings highlight the importance of structural and contextual features of the learning environment, including small class sizes and discussion-based formats. Participants consistently identified peer interaction and psychological safety as central to their learning experience, indicating that the instructional context plays a critical role in shaping outcomes. This perspective extends existing research by positioning classroom structure as an active component of mental health promotion rather than a neutral delivery mechanism. These findings align with broader literature demonstrating that academic stress is a pervasive driver of student distress and that effective interventions must address both individual coping strategies and the educational environments in which students learn [28].

### The Kolb Experiential Learning in Practice

Students’ accounts reveal an iterative learning spiral that closely mirrors the Kolb 4-mode cycle [23,24]. Experiential learning assignments provided concrete experience; guided journals and peer dialogue fostered reflective observation; minilectures on stress physiology or cognitive restructuring offered abstract conceptualization; and the following week’s assignments invited active experimentation. Progressively, learners refined tactics for sleep hygiene, emotion regulation, and time management, demonstrating the Kolb premise that knowledge is continuously rebuilt as experience is tested, reflected upon, and reapplied [23,24].

### The Mezirow Transformative Phases: From Skill to Meaning Change

While Kolb explains how learning moves, many participants described a deeper shift: questioning long-held beliefs, such as *success equals constant busyness*, and embracing new self-definitions (“I schedule rest with purpose”) [25,26]. These narratives align with the Mezirow transformative learning theory: early assignments acted as disorienting dilemmas, prompting critical self-examination; dialogue enabled recognition of shared struggle; and students then explored options, planned actions, tried new roles, and reintegrated reshaped worldviews into daily life [25,26]. Thus, the experiential spiral sometimes culminated not merely in competence but in genuine perspective transformation.

### The R^3^ Cycle: A Pedagogical Bridge

The R^3^ Cycle offers a concise synthesis of these findings, capturing the iterative process through which students move between self-awareness, pattern recognition, and intentional behavior change. Positioned alongside Kolb and Mezirow, the model highlights how experiential learning in this context extends beyond skill acquisition to include meaning-making and identity development. Table 3 provides a practical illustration of the model.

### Contributions to the Wellness-Education Literature

This study advances the wellness-course literature in three key ways. First, it demonstrates that experiential learning curricula can support both discrete coping skill development and deeper shifts in meaning perspectives—outcomes that are often examined separately. Students not only reported acquiring practical strategies (eg, mindfulness, time management, and cognitive restructuring) but also described changes in how they conceptualized success, rest, and self-worth, bridging skill-based and transformative learning perspectives. Second, the R^3^ model offers a concise, practice-oriented framework for understanding how experiential learning translates into both behavioral and meaning-level change. Distilling complex transformational processes into 3 iterative phases allows the proposed model to suggest a structure that is adaptable to educational settings and amenable to operationalization in future research [25,26]. Third, the findings suggest that motivational and contextual factors (particularly experiential practice, reflection, and peer interaction) may help sustain the integration of new skills and perspectives over time. These insights extend existing learning theory by highlighting mechanisms that support persistence beyond the classroom [23-26]. Thus, these contributions can help shift the focus from whether wellness courses are effective to how and why they facilitate meaningful and sustained change in student well-being.

### Limitations

These findings should be interpreted cautiously. First, they derive from a self-selected group at a single faith-based private university; respondents may differ from nonparticipants, and the campus culture, small class sizes, and elective credit structure may limit transferability. Second, the survey instrument was developed for the purposes of this study and was not formally pilot tested or psychometrically validated, which may affect the consistency and comparability of responses. Third, reliance on a single, postcourse open-ended survey limited opportunities for probing, introduced potential social desirability and recall bias, and provided limited evidence of sustained change over time. Fourth, all data were self-reported; objective indicators (eg, physiological stress measures or behavioral logs) were not collected to corroborate perceived gains. Fifth, the sample was predominantly female and non-Hispanic White, which may limit the transferability of findings to more diverse student populations and global nursing contexts, where experiences of stress, coping, and well-being may differ across sociocultural settings. Finally, although purposive sampling enabled the capture of diverse experiences across course types, the sample was drawn from a single institution and may not be fully transferable to other student populations or educational contexts. Together, these limitations underscore the need for mixed methods, multisite, and longitudinal research before broader generalization of the R^3^ model or causal inferences can be made.

### Implications for Health Profession Education

As universities face rising levels of student stress and increasing demand for mental health services, these findings suggest that wellness courses may serve as a scalable, curriculum-based approach to supporting student well-being. Participants reported meaningful gains in self-awareness, coping skills, and behavior change, indicating that such courses extend beyond knowledge acquisition to support personal growth. Given that only 45.5% of students rate their mental health as very good or excellent, and 41.7% identify stress as a barrier to academic success, the integration of wellness education into undergraduate curricula represents a timely and necessary response [8]. These courses may provide upstream support that could help alleviate pressure on campus counseling services. Such implications may also contribute to a broader institutional shift in viewing universities not only as academic environments but also as settings that shape lifelong well-being [27-29]. The findings of this study are consistent with calls for integrating wellness and trauma-informed approaches into nursing education, as existing curricula often lack structured preparation for managing stress and emotional burdens [30]. Wellness courses, therefore, represent both an educational innovation and a promising public health strategy.

The findings also offer several practical implications for educators in nursing and other health professions. First, experiential wellness strategies can be integrated into existing curricula without requiring the addition of standalone courses. Brief, structured activities such as reflective journaling, guided discussion, and applied skill practice (eg, stress regulation techniques) can be embedded within existing coursework to support student well-being. Second, instructional format plays a critical role in shaping student engagement. Small-group and discussion-based learning environments appear to enhance psychological safety, peer connection, and participation. Even within larger programs, incorporating periodic interactive or small-group elements may strengthen learning outcomes. Third, students consistently identify practical, immediately applicable skills as the most impactful. Incorporating content such as stress physiology, cognitive strategies, and time management into health professions education may support both academic success and professional resilience. These approaches align with the growing emphasis on prevention, self-regulation, and well-being in health care training [31]. Fourth, repetition and sustained practice emerge as essential for behavior change. Rather than relying on one-time interventions, wellness education may be more effective when delivered longitudinally through repeated, low-intensity experiential activities that support habit formation over time. Finally, these approaches are feasible within common institutional constraints. Many strategies identified (eg, reflection, peer dialogue, and applied exercises) require minimal financial investment and can be implemented without specialized infrastructure. This positions experiential wellness education as a scalable, curriculum-integrated approach to supporting student well-being in nursing and other health profession programs.

### Conclusions

Experiential wellness courses offer a promising approach to integrating well-being into higher education. In this study, students described meaningful changes in coping, behavior, and self-awareness and perceived course content as relevant and applicable to their lives. These findings may provide insight into how students perceive, engage with, and interpret experiential wellness education. Such courses may highlight the value of combining psychoeducation, applied practice, reflection, and peer dialogue within academic settings. In the context of rising student distress, curriculum-integrated approaches may be considered a complementary strategy to support student well-being.

## Abbreviations

R^3^: reflect-recognize-reorient
